# Preparation and Tribological Properties of WS_2_ Hexagonal Nanoplates and Nanoflowers

**DOI:** 10.3390/nano9060840

**Published:** 2019-06-01

**Authors:** Xianghua Zhang, Jiangtao Wang, Hongxiang Xu, Heng Tan, Xia Ye

**Affiliations:** 1 School of Mechanical Engineering, Jiangsu University of Technology, Changzhou 213001, China; jxxhx@jsut.edu.cn (H.X.); tanheng@jsut.edu.cn (H.T.); yexia@jsut.edu.cn (X.Y.); 2 School of Materials Science and Engineering, Jiangsu University of Technology, Changzhou 213001, China; jxwjt@jsut.edu.cn

**Keywords:** WS_2_, lubricant additives, tribological properties

## Abstract

This paper presents the facile synthesis of two different morphologies of WS_2_ nanomaterials—WS_2_ hexagonal nanoplates and nanoflowers—by a sulfurization reaction. The phases and morphology of the samples were investigated by X-ray diffraction (XRD), scanning electron microscopy (SEM), and transmission electron microscopy (TEM). The tribological performance of the two kinds of WS_2_ nanomaterials as additives in paraffin oil were measured using a UMT (Universal Mechanical Tester)-2 tribotester. The results demonstrated that the friction and wear performance of paraffin oil can be greatly improved with the addition of WS_2_ nanomaterials, and that the morphology and content of WS_2_ nanomaterials have a significant effect on the tribological properties of paraffin oil. The tribological performance of lubricating oil was best when the concentration of the WS_2_ nanomaterial additive was 0.5 wt %. Moreover, the paraffin oil with added WS_2_ nanoflowers exhibited better tribological properties than paraffin oil with added WS_2_ hexagonal nanoplates. The superior tribological properties of WS_2_ nanoflowers can be attributed to their special morphology, which contributes to the formation of a uniform tribo-film during the sliding process.

## 1. Introduction

In recent years, the global energy crisis and environmental pollution have been serious problems. Now, the regulatory requirements for reducing energy consumption and avoiding energy losses are becoming more stringent. Because of this, reducing energy consumption and greenhouse gas emissions has become an important focus for researchers. According to recent research by Holmberg et al., the friction of engines, gearboxes, tires, auxiliary equipment, and brakes in heavy vehicles consumes 33% of fuel energy [1], friction in cars consumes 28% of fuel energy [2], and the energy consumed by internal friction in the entire paper mill accounts for 15–25% [3]. Therefore, many attempts have been made to introduce various methods to overcome friction. Lubrication is known to be one of the most effective ways to reduce friction and wear, and the antifriction effect of lubricating oil is mainly affected by the lubricant additive. Recent studies have found that some nanomaterials have good antifriction performance due to their special structure. Therefore, increasing attention is now being paid to the use of nanomaterials as lubricant additives to improve the tribological properties of lubricating oil.

In the past few years, a variety of nanomaterials have been used as lubricant additives, and their tribological properties have been extensively studied. These materials can be classified into the following categories. The first type is metallic nanoparticles, including Cu, Fe, Ni, etc. [4,5,6]. The second includes carbon materials such as carbon nanotubes and graphene [7,8,9,10]. The third is composed of the transition metal chalcogenides, containing MoS_2_, WS_2_, MoSe_2_, WSe_2,_ etc. [11,12,13,14,15]. The last category comprises other nanomaterials such as oxides, fluorides, and borides [16,17,18,19,20]. Among these different types of materials, transition metal chalcogenides have received great attention due to their special layered structure.

WS_2_, as an important member of the transition metal chalcogenide material family, has attracted great attention for its intriguing electronic, electrochemical, and electrocatalytic properties, and for its extensive applications in field-effect transistors, energy storage, catalysis, and hydrogen storage media [21,22,23,24,25,26,27,28]. In addition, WS_2_ is an excellent solid lubricant due to its special layered structure, which is composed of strong S–W–S covalent bonds inside the layers, and the weak van der Waals force between the layers. The easy sliding between WS_2_ layers under small shear forces is often regarded as an important feature of its excellent lubricity [29]. Recently, WS_2_ nanomaterials with different morphologies have been synthesized, and their tribological properties and antifriction mechanisms have been studied. For example, Tenne et al. [11] investigated the tribological properties of fullerene-like WS_2_ nanoparticles as additives in a lubricating oil under harsh conditions, and the results showed that WS_2_ nanoparticles play a major role in alleviating friction and wear. Wu et al. [30] synthesized hollow WS_2_ spheres by a solvothermal process and compared their tribological properties with commercial colloidal MoS_2_ as an additive in liquid paraffin. Zhang et al. [12] prepared WS_2_ nanorods by using a self-transformation process and investigated the tribological performance of WS_2_ nanorods as an additive in lubricating oil. It was found that the antiwear ability of the base oil was improved by the addition of WS_2_ nanorods. Hu et al. [31] studied the tribological properties of WS_2_ and WS_2_/TiO_2_ nanoparticles dispersed in diisooctyl sebacate and found that the two nano-additives slightly affected the friction reduction effect, but WS_2_/TiO_2_ nanoparticles were found to remarkably improve the wear resistance of diisooctyl sebacate.

All of the above studies have shown that WS_2_ nanomaterials with different morphologies help to improve the tribological properties of lubricating oils. However, these studies have only investigated the tribological properties of WS_2_ nanomaterials with a single morphology, and did not explore the antifriction properties and mechanisms of WS_2_ nanomaterials with a different morphology under the same working conditions. Previous studies investigating MoS_2_ nanomaterials have demonstrated the complex relationship between the morphology size and tribological properties of MoS_2_. For example, Xu et al. reported that the lubricity of the sheet-like nano-MoS_2_ is inferior to that of the micro-scale MoS_2_ in rapeseed oil [32]. However, Raboso et al. reported that the size and morphology of MoS_2_ did not have a significant effect on the friction and wear of the polyalphaolefin oil [33]. Therefore, it is valuable to study the tribological properties and friction reduction mechanism of WS_2_ nanomaterials with different morphologies.

In this study, two different morphologies of WS_2_ nanomaterials—WS_2_ hexagonal nanoplates and nanoflowers—were synthesized by a different high-temperature solid-phase reaction process. The tribological properties of the two kinds of WS_2_ nanomaterials as additives in the paraffin oil were also investigated.

## 2. Materials and Methods

### 2.1. Reagents and Materials

Tungsten and sulfur powders were purchased from Sinopharm Chemical Reagent Co. Ltd (Shanghai, China). Tungsten trioxide and thiourea were obtained from the Aladdin Chemical Reagent Company (Shanghai, China). All chemical reagents were used directly without further purification.

### 2.2. Synthesis of WS_2_ Hexagonal Nanoplates

In a typical method, high-purity tungsten and sulfur powder (W:S molar ratio of 1:3, S powder excess 50%) were poured into a steel kettle, and then the powders were mechanically ground with a speed of 300 rpm (rotations per minute) in a planetary ball mill for 12 h. Then, the ball-milled mixture was transferred into a stainless-steel reactor. The reactor was tightly closed and pushed into the middle of a tube furnace. The temperature of the tube furnace was raised to 650 °C at a rate of 5 °C min^−1^ in an atmosphere of N_2_ and the temperature was maintained at 650 °C for 2 h. Subsequently, the tube was gradually cooled to room temperature and the prepared powders were then obtained.

### 2.3. Synthesis of WS_2_ Nanoflowers

The WS_2_ nanoflowers were synthesized according to the previous method [34] reported by us with minor modifications. This included 10 mmol of WO_3_, 60 mmol of sulfur powder and 140 mmol of thiourea were ground in a mortar for 30 min. Then 3 g of the ground mixture was loaded in an alumina boat. This boat was pushed into the hot zone of the tube furnace. The furnace temperature was maintained at 850 °C for 1 h in N_2_ atmosphere and then gradually cooled to room temperature.

### 2.4. Materials Characterization

The X-ray diffraction (XRD) pattern was recorded by a Shimadzu LabX XRD-6000 X-ray diffractometer using a Cu Ka X-ray source operating at 40 kV and 30 mA with a scanning range of 10° to 80°. A JSM-7001F field-emission scanning electron microscope (FESEM) and a JEM-2100 transmission electron microscope (TEM) were used to record the sample morphology.

### 2.5. Tribological Properties Test

A UMT-2 tribotester (CETR, San Jose, CA, USA) was used to measure the tribological properties of the two WS_2_ samples. The prepared WS_2_ powders were dispersed into the paraffin oil by ultrasonic dispersion for 60 min which then resulted in the required lubricating oil with different WS_2_ contents. The tribological properties tests were performed in ball-and-disk mode with a load of 10–60 N and a rotational speed of 100–400 rpm for 30 min. The friction pair consisted of a ball with a diameter of 10 mm and a disc with size of Φ40 mm × 3 mm. The fixed upper sample (ball) is made of GCr15 bearing steel (AISI 52100) with a hardness of 62 HRC (Rockwellhardness) and the rotating lower sample (disk) is made of 45# steel. The surface of the steel disc was polished and cleaned with acetone before the test. The friction coefficient was automatically recorded during the contact friction, and the widths of the wear scars were measured by an optical microscope. The morphologies and elements of the wear scars on the surface of the lower disc were investigated by scanning electron microscopy (SEM) and energy-dispersive X-ray spectroscopy (EDS).

## 3. Results and Discussion

### 3.1. Structure and Morphology Characterization

**Figure 1:**
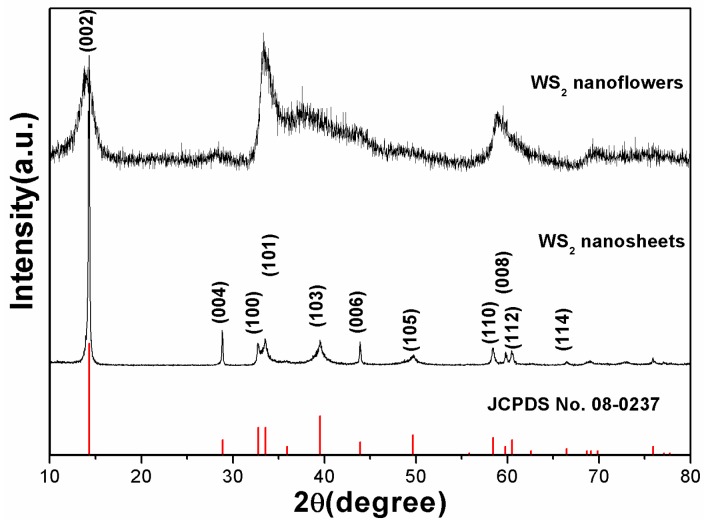
X-ray diffraction (XRD) pattern of the as-synthesized WS_2_ hexagonal nanoplates and nanoflowers.

The crystal structure and phase purity of the synthesized samples were verified by the XRD patterns, as presented in Figure 1. From the image, it can be seen that the diffraction patterns of the two samples were significantly different. The diffraction peaks of the nanoplates located at 14.30°, 28.84°, 32.74°, 33.50°, 39.50°, 43.90°, 49.70°, 58.40°, 59.80°, 60.48°, and 66.50° were assigned to the (002), (004), (100), (101), (103), (006), (105), (110), (008), (112), and (114) planes of WS_2_, respectively. Furthermore, a high and sharp (002) peak was observed from the XRD pattern, indicating that the WS_2_ nanoplates were stacked together with a highly ordered packing [35]. In contrast, only (002), (100), (101), and (110) peaks could be detected in the diffraction pattern of the nanoflower sample. Besides, the intensity of the (002) peak located at 13.76° was significantly weakened, and its position was shifted to the left by 0.56° from the standard card. This indicates that the number of stacks in the (002) layer of the WS_2_ nanoflowers was reduced, and the layer interval became larger [36]. All the diffraction peaks of the two patterns could be indexed to the hexagonal phase (p63/mmc space group) of WS_2_ (JSPDS No. 08-0237). No evidence of any other phases was detected, indicating that the samples were of high purity.

**Figure 2:**
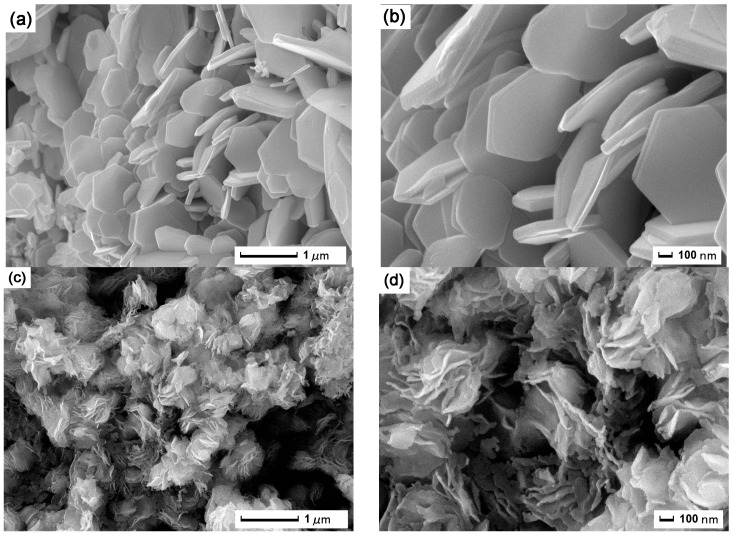

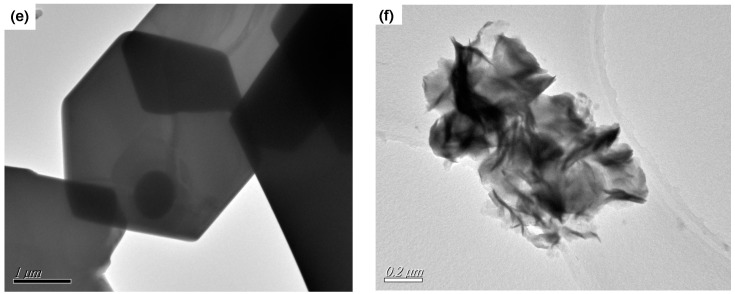
Scanning electron microscopy (SEM) images of WS_2_ hexagonal nanoplates (**a**,**b**) and nanoflowers (**c**,**d**); transmission electron microscopy (TEM) images of WS_2_ hexagonal nanoplates (**e**) and nanoflowers (**f**).

The morphology and size of the two fabricated WS_2_ samples were identified by SEM and TEM. The SEM images of the WS_2_ hexagonal nanoplates are presented in Figure 2a,b. From the low-magnification SEM image (Figure 2a), it can be seen that the sample was composed of a large number of regular nanoplates with the diameter of about 0.5–1 μm. The SEM image with higher magnification in Figure 2b presents a clear view of the surface morphology of the nanoplates. These nanoplates exhibited hexagonal morphology with a thickness of 50–100 nm. Figure 2c,d displays the SEM images of the WS_2_ nanoflowers. Some agglomerated WS_2_ flower-like structures are presented in Figure 2c. It can be seen from the enlarged image (Figure 2d) that these nanoflowers were composed of some ultrathin nanosheets, and the edges of these nanosheets were obviously curled. That is because these nanosheets are unstable and tend to form a closed structure by rolling up, thereby reducing the number of dangling bonds and the total energy of the system [37].

To further reveal the morphology and microstructure of these WS_2_ nanomaterials, TEM measurements were performed on the samples. As shown in Figure 2e, perfectly hexagonal WS_2_ nanoplates with diameters of 3.5 μm were observed. The TEM image of the WS_2_ nanoflower is shown in Figure 2f, from which it can be seen that the WS_2_ nanoflowers were dispersed into ultrathin nanosheets after sonication, but the nanosheets were still connected together. In addition, the edges of the nanosheets were significantly curled, which is consistent with the SEM photographs.

### 3.2. Analysis of Tribological Properties

**Figure 3:**
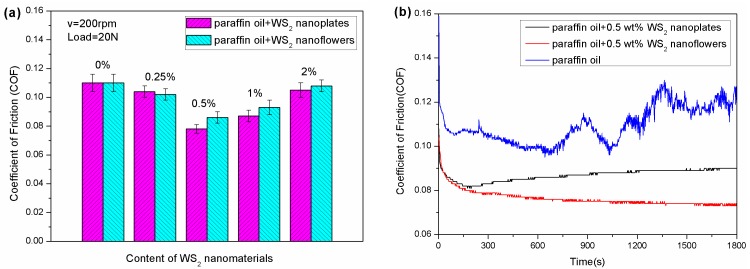
(**a**) The changes of the average friction coefficient of the WS_2_ nanoplates and nanoflowers with different concentration, and (**b**) the real-time friction coefficient as a function of sliding time when lubricated by three different oil samples.

The tribological properties of the two different WS_2_ nanomaterials as lubricant additives in paraffin oil were investigated by a UMT-2 tribotester. Figure 3a shows the effect of the nanomaterial additive concentrations on the tribological properties with a working load of 20 N at 200 rpm for 30 min. From this, it could be found that the average friction coefficient of paraffin oil with the addition of the two different nanomaterials was smaller than that of pure paraffin oil. When the content of the additives was 0.5 wt %, the friction coefficient of the paraffin oil with WS_2_ nanoflowers reached the lowest value, a reduction of 29.1% in comparison with pure paraffin oil, while that of paraffin oil containing WS_2_ nanoplates was only reduced by 24.5%. Additionally, when the concentration of the nanomaterials was higher than 0.5 wt %, the friction reducing performance was gradually weakened with the increase of the additive concentration. It can be concluded that the friction coefficient will increase when the additive content is too low or too high. The reason is that when the concentration is too low, a continuous lubricating film cannot be formed on the surface of the friction pair, and when the concentration is too high, the additive will agglomerate, which affects the friction-reducing effect [38]. Figure 3b exhibits the real-time friction coefficient curve of pure paraffin oil and the two lubricating oils with 0.5 wt % nanomaterial added. In the beginning, the three curves had the same trend, and the friction coefficient changed from large to small, which is attributable to the lack of lubricant between the friction pairs. With the embedding of the lubricant, the friction coefficient was drastically reduced. However, after 10 min, the friction coefficient of the pure paraffin oil began to increase, and the coefficient fluctuated greatly. However, the friction coefficient of the paraffin oil with added nanomaterials was very stable, and the friction coefficient of the WS_2_ nanoflowers was always lower than that of the nanoplates. The above experimental results indicate that the paraffin oil containing WS_2_ nanoflowers possessed better lubricating properties than both the pure paraffin oil and the paraffin oil containing WS_2_ nanoplates.

**Figure 4:**
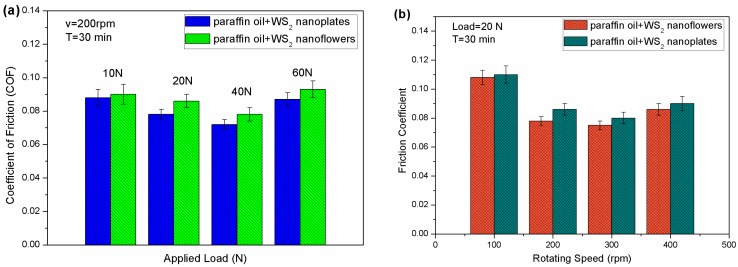
The changes of average coefficient of friction of the WS_2_ nanoflowers and nanoplates with different load (**a**) and different rotating speed (**b**).

In order to further compare the tribological properties of the two WS_2_ nanomaterials, comparative experiments were carried out with different loads and different rotating speeds. Figure 4a shows the average friction coefficient as a function of applied load when the additive concentration was 0.5 wt % and the tribotester was operated with a rotating speed of 200 rpm for 30 min. Obviously, when the applied load was increased from 10 to 40 N, the average friction coefficient had a downward trend. However, when the load was increased to 60 N, the coefficient increased slightly. The variation of the average friction coefficient of the two kinds of WS_2_ nanomaterials with the change of rotating speed is presented in Figure 4b. The trend of the average friction coefficient in Figure 4b is similar to that in Figure 4a, which indicates that the friction coefficient first decreased with increasing speed, and then increased. In addition, under the same conditions, the average friction coefficient of the WS_2_ nanoflowers was always lower than that of the WS_2_ nanoplates.

From the above results, it was found that WS_2_ nanoflowers exhibited better tribological properties, and it was found that the friction coefficient could be remarkably decreased by adding two kinds of WS_2_ nanomaterials into the paraffin oil.

**Figure 5:**
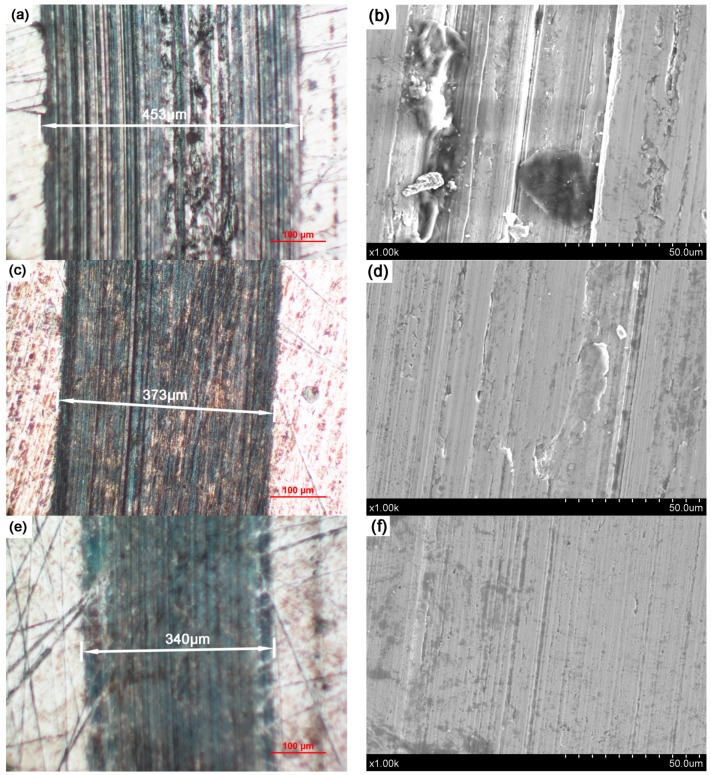
Optical images and SEM micrographs of wear scars lubricated with pure paraffin oil (**a**,**b**), paraffin oil + 0.5 wt % WS_2_ nanoplates (**c**,**d**), and paraffin oil + WS_2_ nanoflowers (**e**,**f**).

In order to compare and analyze the antiwear properties of the two kinds of WS_2_ nanomaterials, the wear surface of the steel disc was examined by optical microscopy and SEM. The optical micrograph and SEM images of the wear surface lubricated with pure paraffin oil, and paraffin oil with 0.5 wt % added WS_2_ nanoplates and nanoflowers are shown in Figure 5. The test time was 30 min and the test load was 20 N. It can be seen from Figure 5a that when the friction pair was lubricated with the pure paraffin oil, the width of the wear scar was about 453 μm. When WS_2_ nanoplates and nanoflowers were added into the paraffin oil, the width of the wear scar was significantly reduced to 373 μm (Figure 5c) and 340 μm (Figure 5e). Furthermore, some deep and wide furrows were observed on the wear surface shown in Figure 5a, which clearly indicates that the surface was subjected to a large contact stress during sliding. The same result was also found in the SEM image. As shown in Figure 5b, many grooves and pits were discovered on the surface of the wear scar, and some abrasive grains with different sizes were attached to it. When 0.5 wt.% of WS_2_ nanoplates were added into the paraffin oil, the surface topography of the wear scars was significantly improved. A dark-colored tribo-film can be observed in the optical picture (Figure 5c), but the tribo-film was unevenly distributed. In the TEM image (Figure 5d), only some very shallow grooves and some very small grinding debris can be found. Compared to the above two lubricants, the friction surface added with the WS_2_ nanoflower lubricant was the least damaged. A large number of dark areas (tribo-film) were observed on the track in Figure 5e and only a few very shallow grooves were seen in Figure 5f and no wear debris was found. These results verify that WS_2_ nanosheets and nanoflowers can improve the antiwear performance of paraffin oil, but the antiwear ability of WS_2_ nanoflowers is better than that of nanoplates.

**Figure 6:**
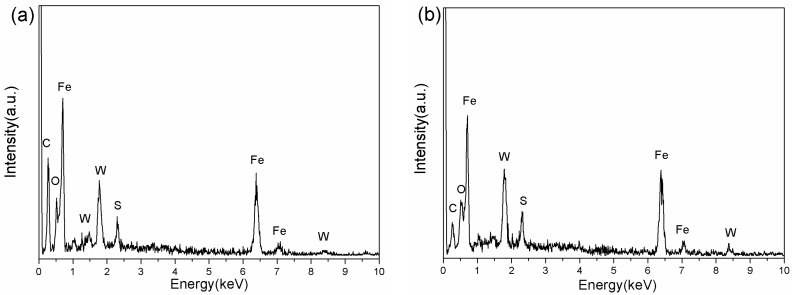
Energy-dispersive X-ray spectroscopy (EDS) of the worn scar of a steel disc lubricated with 0.5 wt % WS_2_ nanoplates (**a**) and nanoflowers (**b**).

In order to investigate the lubrication mechanism of the WS_2_ nanoplates and nanoflowers, EDS was used to investigate the worn surface. The EDS spectra obtained from the worn scar of Figure 5d,f are presented in Figure 6. The elements of W and S were present on the worn surface. This could prove that there was WS_2_ deposited on the worn surface during the process of friction.

There is a great deal of literature regarding the antifriction and antiwear mechanisms of nanomaterials as lubricant additives, and they can be summarized into the following three reasons. The first is that the nanomaterial produces a rolling effect on the surface of the friction pair [12]. The second reason is that the nanomaterial adsorbed on the surface of the friction pair forms a lubricating film [13]. The last reason is that nanomaterials have a repair effect on the surface of the friction pair [4,6].

According to the above experimental results, we could infer the reasons for the friction reduction and antiwear properties of the WS_2_ nanoplates and nanoflowers. The main reason can be attributed to the formation of a tribo-film on the rubbing surface, but there was still a difference in the mechanism of the antifriction and antiwear between the WS_2_ nanoplate and the nanoflower at the beginning. When the WS_2_ nanoplates were used as lubricant additive, the WS_2_ nanoplates would penetrate into the interface of the friction surface. However, due to the large thickness of the nanoplates, they could not be firmly adsorbed on the surface of the friction pair. Due to the layered structure of the nanoplate, some thin nanosheets would be peeled off from the nanoplates during the continuous extrusion process by the friction pair. These stripped nanosheets would be adsorbed on the surface of the friction pair and then form a lubricating film. However, due to the different thickness of the stripped nanosheets, the resulting lubricating film was uneven. In contrast, after ultrasonic dispersion, WS_2_ nanoflowers were decomposed into some ultrathin nanosheets, as demonstrated by the TEM image in Figure 2f. We have researched the antifriction and antiwear mechanism of the ultrathin WS_2_ nanosheets as additives in 500 SN base oil [38]. The antifriction mechanism of the WS_2_ nanoflowers and the WS_2_ ultrathin nanosheets is the same. When the nanoflowers were dispersed into nanosheets, the dispersed nanosheets quickly adhered to the surface of the friction pair and formed a lubricating film, further reducing the wear on the surface of the friction pair. Since the thickness of the ultrathin nanosheets forming the nanoflowers is substantially the same, when the nanoflowers are decomposed, a tribo-film with uniform thickness is formed. The uniform tribo-film can improve tribological performance. Therefore, WS_2_ nanoflowers as a lubricant additive have better antifriction and antiwear properties than WS_2_ nanoplates.

## 4. Conclusions

In this study, WS_2_ hexagonal nanoplates and nanoflowers were successfully synthesized by a solid-phase reaction. Tribological tests demonstrated that the tribological properties of paraffin oil could be greatly improved with the addition of the two kinds of WS_2_ nanomaterials, and the morphology and content of the WS_2_ nanomaterials had a significant effect on the tribological properties of paraffin oil. The optimum nanomaterial concentration was 0.5 wt %. The paraffin oil with added WS_2_ nanoflowers exhibited better friction reducing and antiwear properties than the WS_2_ hexagonal nanoplates. With the addition of the WS_2_ nanoflowers, the friction coefficient was stably maintained at a low value and the wear surface appeared to be smoother. The superior tribological performance of WS_2_ nanoflowers can be attributed to their special structure. Since the nanoflowers are decomposed into a number of ultrathin nanosheets, and these nanosheets are adsorbed on the surface of the friction pair which forms a uniform tribo-film, this can reduce friction and wear.
